# A pilot study on integrating mindfulness-informed professional development for EFL teachers

**DOI:** 10.3389/fpsyg.2026.1771786

**Published:** 2026-06-11

**Authors:** Oya Pelin Bektaş, Merve Geçikli

**Affiliations:** 1 Yunus Emre Secondary School, Bayburt, Türkiye; 2 Department of English Language Teaching, Atatürk Üniversitesi, Erzurum, Türkiye

**Keywords:** EFL pedagogy, EFL teachers, emotional regulation, mindfulness, mindfulness-informed professional development, teacher mindfulness, teacher wellbeing

## Abstract

This pilot mixed-methods case study investigates the mindfulness levels and perceptions of secondary school EFL teachers as the initial phase of a larger intervention project aimed at enhancing teacher wellbeing and professional competence. Acknowledging the emotional, cognitive, and relational demands of language teaching, this study assesses teachers’ mindfulness skills and examines conditions that influence their openness to and engagement with mindfulness-based practices today. Thirty-one teachers completed two standardized instruments—the Mindfulness in Teaching Scale (MTS) and the Kentucky Inventory of Mindfulness Skills (KIMS)—while eight teachers participated in semi-structured interviews. Quantitative findings showed moderate overall mindfullness, with higher competence in observing and describing and lower performance in acting with awareness and accepting without judgment, while observing emerged as the strongest predictor of overall mindfulness. Qualitative results revealed limited prior knowledge of mindfulness, but generally positive attitudes toward its potential benefits for emotional regulation, stress reduction, and classroom climate. Teachers also identified several barriers to practical implementation, including time constraints, insufficient training, institutional pressures, and possible misconceptions among administrators or parents. Despite these concerns, participants expressed interest in professional development opportunities that provide clear conceptual grounding, practical strategies, and classroom-relevant applications. Taken together, these findings underscore the need for mindfulness-informed professional development that systematically builds mindfulness skills through scaffolded, context-sensitive approaches. The study provides a foundational evidence base for designing a targeted mindfulness-based intervention intended to strengthen EFL teachers’ wellbeing, resilience, and pedagogical presence within demanding instructional environments.

## 1 Introduction

The growing recognition of mindfulness as a valuable educational and psychological construct has generated increasing interest in its integration into teacher education, particularly within English as a Foreign Language (EFL) contexts, where teachers face substantial emotional, cognitive, and instructional demands. Teaching English requires not only pedagogical expertise but also emotional regulation, interpersonal sensitivity, and adaptive decision-making under institutional constraints and diverse learner needs (Hatton-Bowers et al., 2020; Hue and Lau, 2015; Nadlifah et al., 2023; Wanders et al., 2020; Zhu, 2022). Within this demanding environment, mindfulness in psychology is commonly defined as a focused, present-centered, and non-judgmental awareness of one’s ongoing experiences and internal states (Buchanan, 2017; Kabat-Zinn, 2005, 2015). In educational contexts, teacher mindfulness extends this concept to the professional domain, referring to teachers’ capacity to remain attentive, emotionally aware, and responsive in their interactions with students and classroom situations (Jennings, 2015; Song and He, 2021). As both a psychological resource and a pedagogical disposition, teacher mindfulness has been associated with enhanced wellbeing, instructional responsiveness, and positive classroom climate (Birchinall et al., 2019; Clarke and Draper, 2020; Corthorn et al., 2024; Dave et al., 2020; Derakhshan, 2022; Flook et al., 2013; Hidajat et al., 2023; Khodabakhshzadeh et al., 2020; Kyte, 2016; Molloy Elreda et al., 2019; Skelly and Estrada-Chichón, 2021; Takiguchi, 2015).

In EFL educational context, extending beyond a general psychological trait, mindfulness can be framed as a situated pedagogical expertise involving awareness of one’s internal states (e.g., emotions, thoughts), sensitivity to learners’ needs, and responsiveness to the evolving classroom context. Previous studies indicate that mindful teachers demonstrate improved teacher–student relationships, greater emotional resilience, and enhanced pedagogical adaptability (Huang, 2022; Tuyan and Kabadayi, 2018; Zhu, 2022). Moreover, through its role in fostering self-awareness and reflective depth, mindfulness has been linked to professional development (PD) (Demir et al., 2025; Weisi and Salari, 2024). Besides, differing from reflective practice, mindfulness focuses on real-time, embodied awareness rather than retrospective, cognitive analysis (Koçali and Asik, 2022) and serves as a foundation for emotional intelligence and self-regulation (Wang, 2022). Thus, mindfulness-informed PD, targeting wellbeing of teachers and so integrating contemplative exercises, reflective activities, and pedagogical applications, involves programs structured around cultivating present-moment awareness, emotional regulation, and reflection within teaching practice (Albrecht et al., 2012; Kuru Gönen, 2022; Roeser et al., 2012; Wang and Liu, 2016).

Existing interventions show positive outcomes, including improved teacher wellbeing, job satisfaction, and self-efficacy, along with better emotional regulation and reduced anxiety, more supportive classroom interactions, suggesting mindfulness enhances both individual and relational aspects of teaching (Cicek and Gurbuz, 2023; Pan and Liu, 2022). Nevertheless, several limitations also persist. Additionally, mixed or uneven results regarding the sustainability of long-term outcomes often stem from inconsistent participant engagement, contextual constraints, variability in program design, and cultural differences (Kuru Gönen, 2022; He et al., 2023; Koçali and Asik, 2022; Zhi and Wang, 2024). These highlight the need for context-sensitive, evidence-based adaptations rather than universal approaches. Furthermore, mindfulness interventions for teachers are informed by diverse theoretical frameworks. Mindfulness-Based Stress Reduction (MBSR) emphasizes non-judgmental awareness to promote stress reduction and wellbeing, demonstrating effectiveness in enhancing resilience and reducing burnout (Kabat-Zinn, 2005; Wang and Liu, 2016). In contrast, cognitive-behavioral approaches integrate mindfulness with strategies to modify maladaptive thinking, linking it to emotional intelligence and professional competence (Xu and Wang, 2024). Other perspectives highlight relational and affective dimensions, focusing on teacher–student interactions (Song and He, 2021). These varied orientations indicate that mindfulness is a multifaceted construct requiring careful theoretical alignment and context-sensitive implementation (Albrecht et al., 2012; DiCarlo et al., 2020; Gold et al., 2010; Gouda et al., 2016; Henriksen et al., 2022; Hoskins, 2020; Lomas et al., 2017; Roeser et al., 2012; Song and He, 2021). Drawing on these complementary theoretical perspectives, the present study adopts a holistic view of mindfulness that considers cognitive, emotional, and relational dimensions in examining EFL teachers’ mindfulness levels, beliefs, and contextual experiences.

Despite the growing interest in mindfulness within teacher education, important conceptual and empirical ambiguities remain, particularly regarding how mindfulness is theoretically defined, operationalized, and translated into EFL teaching practice across different sociocultural and institutional contexts. Existing studies draw on diverse and sometimes conflicting frameworks—ranging from stress-reduction and cognitive-behavioral perspectives to relational and affective approaches—resulting in inconsistent understandings of what constitutes teacher mindfulness and how it should be implemented in professional development. Empirically, the literature is also limited by a reliance on short-term intervention studies, insufficient attention to teachers’ contextual realities and institutional constraints, and a lack of research examining teachers’ prior knowledge, readiness, and perceptions before mindfulness-based interventions are introduced, particularly in underexplored EFL settings. Consequently, further research is needed to clarify how mindfulness is understood and enacted in specific EFL contexts, as well as to examine teachers’ readiness, beliefs, and contextual experiences prior to intervention, thereby informing the design of contextually appropriate and pedagogically feasible mindfulness-informed professional development programs. Addressing this gap, this pilot mixed-methods case study investigates teachers’ mindfulness levels, beliefs, and contextual barriers to engagement. In this regard, the study contributes to the field of language teacher education by offering empirically grounded insights that can inform the development of responsive teacher education and professional development initiatives in EFL settings. Accordingly, the study is guided by the following research questions:

1. What are the levels and dimensions of mindfulness among EFL teachers in the target context?
2. What beliefs, attitudes, and experiences do EFL teachers hold regarding mindfulness practices?
3. What contextual and individual factors facilitate or hinder teachers’ engagement with mindfulness?
4. How can these findings inform the design of a context-sensitive mindfulness-informed professional development program?

## 2 Methods

### 2.1 Research design

This study adopted an explanatory sequential mixed-methods case design, in which quantitative data were collected and analyzed first, followed by qualitative data to further explain the quantitative findings. This approach enabled a comprehensive understanding of EFL teachers’ mindfulness levels, perceptions, and contextual experiences by integrating quantitative and qualitative methods. Findings from the MTS and KIMS scales guided the selection of interview participants and the development of the qualitative phase, while triangulation through the integration of quantitative results and qualitative themes enhanced the depth and credibility of the interpretation.

### 2.2 Participants

The target population for this study consisted of secondary school EFL teachers working in K–12 public schools across Türkiye’s East Anatolian Region, where approximately 410,000 learners receive English language instruction. A purposive convenience sample was drawn from volunteers who met the study’s selection criteria. Teacher recruitment was conducted through teachers’ listservs where the information about the study and an online participation link (posted with permission) were circulated. Teachers who agreed to join in the present study filled out web-based scales through a Google Form, which asked demographic questions and measured levels of mindfullness. Thirty-one EFL teachers were ultimately recruited for the planned intervention phase, which aligns with methodological recommendations introduced by Kunselman (2024) to measure Mindfulness-informed professional development. The participant group consisted predominantly of young, early-career teachers (between 5 and 10 year experience) with no prior mindfulness experience at all, with most being female (83.9%), aged between 23 and 30 (71%), and holding a bachelor’s degree (97.4%), while a smaller proportion were male (16.1%), older (31–42), or held a master’s degree (2.6%).

The decision to include this group in both the first pilot phase and forthcoming intervention stages was based on MTS outcomes and the physical conditions of educational settings they are working in. The results indicated that respondents had intermediate level of mindfulness in general. Teachers reported relatively strong abilities in observation and emotional awareness, reflected in high mean scores for noticing bodily sensations (*M* = 4.00) and articulating emotions (*M* = 4.35). However, scores for maintaining awareness in daily teaching tasks (routine) were below the average (*M* < 3.50), which suggests difficulties with being mindful in general day-to-day instructional activities. While teachers showed good observational and descriptive skills, they performed poorly for sustained attention and emotional acceptance. Moreover, this group works in varied educational settings with differing structural and physical conditions with class sizes ranging from 15 to 40, limited weekly English lesson hours —2 h at the primary level and 3–4 h at the lower secondary level, notable disparities in school facilities—some classrooms with smart boards, others lack basic technological resources, scarce language laboratories not sufficiently supported with instructional materials, which shape the working conditions of EFL teachers and contribute to increased workload and instructional challenges. Both MTS outcomes and these contextual constraints emphasize the importance of targeted professional development focused on enhancing intentional attention and emotional regulation across different teaching environments.

In the pilot’s second, qualitative phase, semi-structured interviews were conducted with eight teachers using purposeful maximal variation sampling—an approach widely used in mixed-methodological designs (Teddlie and Tashakkori, 2009). Participants were further classified into lower, moderate and higher levels of mindfulness based on their quantitative scores in all dimensions (from KIMS and MTS), differentiated by their strongest mindfulness dimension: observing, describing, acting with awareness, and acting without judgment (see Table 1). This method allowed for the coverage of a wide range of mindfulness profiles and teaching experience. The diversity of cases added richness to the qualitative narratives by giving detail as to how teachers conceptualize mindfulness, what it means for them in their professional lives, and what challenges they ran into with respect to implementing mindfulness practices. Together, these insights explicitly shaped the framework of the forthcoming mindfulness-informed professional development intervention.

**TABLE 1:** Characteristics of interviewees.

Interviewee	Year of experience	Mindfulness level	Mindfulness dimension
1	5 year	Lower	Observing
2	9 year	Lower	Observing
3	10 year	Lower	Describing
4	10 year	Lower	Describing
5	7 year	Moderate	Acting with awareness
6	8 year	Moderate	Acting with awareness
7	8 year	Moderate	Accepting without judgment
8	9 year	Higher	Accepting without judgment

### 2.3 Measures and procedure

Before the research, ethical approval was received from respective institutional review board and all teachers provided informed consent following the guidelines of ethical considerations in research. Subsequently, English teachers were recruited and MTS was conducted to determine the levels of mindfulness as a first step. Developed by Frank et al. (2016), the scale has two factors “teacher intrapersonal mindfulness” (9 items) and “teacher interpersonal mindfulness” (5 items) and consists of items presented on a five-point Likert scale, ranging from Never true to Always true. For the current study, the internal consistency coefficients of the scale demonstrated strong reliability, ranging from 0.78 to 0.93 (Field, 2018), supporting the production of valid and trustworthy findings (DeVellis, 2017). In addition, the scale has been widely used in educational and mindfulness research, demonstrating strong construct validity and suitability for assessing teachers’ mindfulness in instructional contexts. Simultaneously, KIMS, developed by Baer et al. (2004), was used to assess four key mindfulness components identified in the literature: observing, describing, acting with awareness, and accepting without judgment. The internal consistency coefficients of the scale ranged from 0.83 to 0.91, supporting the scale’s capacity to yield accurate and valid results (Baer et al., 2004). Furthermore, KIMS has been empirically validated across diverse populations and has shown strong psychometric properties, making it an appropriate instrument for measuring multidimensional mindfulness constructs in this study. The use of both scales was intended to provide a complementary assessment of teachers’ mindfulness by capturing both context-specific teaching-related mindfulness and its underlying multidimensional skills, thereby enabling a more comprehensive examination of how general mindfulness competencies relate to and support mindfulness in instructional practice.

In the second part of the study, semi-structured interviews with the eight teachers were performed. The semi-structured interview questions were developed by the researchers based on the key notions of mindfulness through relevant literature and expert opinions in a systematic manner through pilot interviews. They attempted to question EFL teachers’ first-hand experience of mindfulness and the depth they have toward understanding and practicing these on their professional life. They also explored the attitudes of teachers toward the function of mindfulness in a classroom and its potential advantages in terms of wellbeing and teaching performance. The interviews also inquired into the needs of professional development particularly related to mindfulness and continued by investigating the type of training or resources which would be helpful to teachers. Each interview took approximately between 1 h and one and a half hours to complete, depending on the depth and pace of participants’ responses.

### 2.4 Data analysis

In the first step of quantitative data analysis, normality tests were conducted to ensure that the data followed a normal distribution. The Shapiro–Wilk test yielded significance levels of 0.488 for the Mindfulness in Teaching Scale (MTS) and 0.596 for the Kentucky Inventory of Mindfulness Skills (KIMS). Since all test results exceeded the 0.05 threshold, indicating normal distribution, and both skewness (MTS = 0.187; KIMS = −0.248) and kurtosis (MTS = −0.476; KIMS = −0.170) values fell within the acceptable -1 to +1 range, the data can be considered normally distributed (Hair et al., 2013). Followingly, descriptive statistics were used to summarize teachers’ performance across mindfulness dimensions, followed by coefficient correlation analysis to examine relationships between overall mindfulness and the four KIMS subscales (Supplementary material). Multiple regression analysis was also conducted to identify which mindfulness components significantly predicted overall mindfulness scores.

In terms of qualitative data, the researchers employed content analysis by examining teachers’ statements to identify common ideas related to their knowledge, experiences, perceptions, benefits, and challenges concerning mindfulness (Creswell and Creswell, 2017). Categories and codes were generated through an inductive content analysis process in which meaningful segments of text were labeled, compared, and clustered according to conceptual similarity before being organized into broader themes. The researchers identified themes by comparing participants’ statements and noting common patterns such as limited prior knowledge, positive attitudes, institutional concerns, and need for training. To ensure the rigor and trustworthiness of the analysis, the strategies such as inter-coder reliability (established among the researchers, with a coefficient above 0.70 showing consistency in coding and interpretation), credibility (strengthened through prolonged engagement with the data and member checking), dependability (supported by maintaining an audit trail of coding and theme development and studying with an external qualitative researcher to minimize bias), and researcher triangulation (achieved through collaborative discussions and consensus) were employed.

## 3 Results

### 3.1 Quantitative results of teachers’ mindfulness skills

Analysis of KIMS revealed that secondary school English teachers exhibited varying levels of proficiency across the four dimensions of mindfulness, with generally moderate performance overall. The observing dimension comprised 12 items, yielding a maximum score of 60. On the other hand, describing skills were measured using 8 items, with a maximum score of 40. The acting with awareness dimension consisted of 10 items, allowing for a maximum score of 50. Finally, the accepting without judgment dimension included 9 items, with a maximum possible score of 45. Accordingly, teachers demonstrated the strongest skills in the observing dimension (*M* = 41/60), indicating above-average attentiveness to internal and external experiences. Their describing skills were also above average (*M* = 30.55/40), suggesting a relatively strong ability to verbalize emotions, thoughts, and sensory experiences. In contrast, teachers scored at a moderate level in the acting with awareness dimension (*M* = 31.39/50), reflecting occasional difficulties with sustaining present-moment focus during tasks. The accepting without judgment dimension yielded the lowest performance (*M* = 26.06/45), revealing challenges in refraining from self-criticism or evaluative thinking about thoughts and emotions. Collectively, these findings indicate that teachers’ mindfulness skills were concentrated in foundational areas (observing and describing), while more complex skills involving acting with awareness and accepting without judgment were less developed.

A correlation analysis was conducted to examine the relationship between secondary school English teachers’ overall mindfulness levels, as measured by the MTS, and the four dimensions of the KIMS (see Table 2). Results indicated a strong, statistically significant positive correlation between overall mindfulness and the observing dimension (*r* = 0.628, *p* < 0.001), suggesting that teachers with higher mindfulness levels were more attentive to internal and external experiences.

**TABLE 2:** Correlations between overall mindfulness and KIMS mindfulness dimensions.

Mindfulness dimensions	n	X	S.s.	*r*	*p*
MTS	31	55.67	6.128	1	
Observing	31	41.48	6.076	0.628	0.000
Describing	31	30.548	4.884	0.469	0.004
Acting with awareness	31	31.38	5.589	0.349	0.027
Accepting without judgement	31	26.06	5.807	-0.043	0.408

A moderate positive correlation was found between mindfulness and the describing dimension (*r* = 0.469, *p* = 0.004), while a weak positive correlation emerged for the acting with awareness dimension (*r* = 0.349, *p* = 0.027), indicating that higher mindfulness contributed only slightly to teachers’ ability to remain focused on present activities. In contrast, the relationship between mindfulness and the accepting without judgment dimension was not significant and near zero (*r* = −0.043, *p* = 0.408), demonstrating no meaningful association. Regression results further supported these findings, showing that the observing skill significantly predicted overall mindfulness, with a strong standardized beta coefficient (β = 0.628, *p* < 0.01) (see Table 3).

**TABLE 3:** Regression analysis predicting overall mindfulness scores.

Variable	Unstandardized coefficients		Standardized coefficients		
	B	SS	β	*t*	*p*
Observing	0.634	0.146	0.628	4.813	< 0.01

### 3.2 Qualitative insights into teachers’ perceptions and challenges

Table 4 presents the coded thematic framework summarizing teachers’ insights related to mindfulness, including the main themes, subthemes, and representative codes identified through qualitative analysis. The qualitative analysis revealed a set of interrelated yet internally complex themes reflecting teachers’ understandings of mindfulness, their attitudes toward its use, and the contextual conditions shaping its feasibility. Rather than presenting uniform perspectives, the findings highlight variation, tension, and contradiction, offering a nuanced picture of teachers’ readiness for mindfulness-informed professional development. As illustrated throughout the data, teachers’ views are not fixed but are shaped through reflection, experience, and context. A dominant finding concerns teachers’ limited ability to define or explain mindfulness. This lack of conceptual clarity appears to stem not simply from individual knowledge gaps but from the broader absence of mindfulness within professional training and discourse. Many participants reported unfamiliarity with the concept and struggled to articulate its meaning without guidance. For instance, one teacher remarked, “*At the moment, I feel unable to generate reasonable thoughts on the topic and tend to avoid making unnecessary contributions*. *(Interviewee 1)*” Importantly, several teachers constructed their understanding during the interview itself, often relying on the researcher’s explanations. This suggests that mindfulness is experienced as an externally introduced construct rather than an embedded professional competency. Therefore, uncertainty should be interpreted as an expected outcome of limited exposure rather than resistance.

**TABLE 4:** Coded thematic framework for mindfulness-related teacher insights.

Theme	Category	Codes
1. Limited prior knowledge and conceptual uncertainty about mindfulness	Lack of exposure	• Never heard of mindfulness • No prior personal or professional experience
	Difficulty articulating the concept	• Unable to define mindfulness • Uncertain about how mindfulness works
	Low conceptual and practical clarity	• Understanding shaped only by interview explanations • Confusion between mindfulness and other practices
2. Teachers’ attitudes and beliefs toward mindfulness	Positive attitudes toward emotional and professional benefits	• Recognize potential for emotional regulation • Believe mindfulness may improve wellbeing
	Skepticism rooted in personal preferences	• Reliance on their own coping strategies • Question the usefulness of adopting new techniques
	Variability tied to personal awareness levels	• Teachers with higher self-awareness show stronger acceptance • Teachers with lower awareness express hesitation
3. Perceived contributions of mindfulness in educational contexts	Stress reduction for students	• Helps students manage stress and emotional load
	Reducing language-learning anxiety	• Supports students who feel embarrassed or anxious during language tasks
	Enhancing focus and emotional regulation	• Improved ability to stay calm and attentive • Useful for emotional control in class
	Benefiting high-stakes exam preparation	• Supports exam-related anxiety • Helps prepare mentally for pressured situations
4. Concerns and challenges in implementing mindfulness	Perceived institutional misunderstandings	• Administrators may misinterpret activities as “not teaching” • Worry about parent misunderstanding
	Classroom management concerns	• Students may struggle to transition back to tasks • Fear of off-task behavior
	Lesson flow disruption	• Mindfulness may break the continuity of lessons • Concern about losing instructional time
5. Barriers to effective implementation	Time constraints	• Insufficient lesson time for mindfulness activities
	Workload intensity	• Heavy workload limits teachers’ ability to incorporate new practices
	Lack of training and knowledge	• No formal preparation or guidance on mindfulness practices
	Need for professional development	• Desire for structured workshops or PD sessions
	Concerns about repetition or misuse	• Fear of overusing the techniques or implementing them incorrectly

Despite this initial unfamiliarity, many teachers expressed positive attitudes once mindfulness was explained, particularly in relation to emotional awareness and regulation. One participant stated, “I *believe it’s a skill that should be integrated into our lives… It’s the ability to recognize and manage our feelings*. *(Interviewee 6)*” Such responses indicate that mindfulness resonates with teachers’ lived emotional realities. However, this positivity was not consistent across all participants. A degree of ambivalence emerged, with supportive perspectives coexisting alongside skepticism. For example, one teacher commented, “*I already have my own relaxation methods… so I don’t really feel the need for something like this… overall, it sounds a bit silly to me*. *(Interviewee 1)*” This perspective reflects resistance grounded not in rejection of the idea itself but in a sense of self-sufficiency and established coping strategies. These contrasting views demonstrate that receptivity is shaped by individual dispositions and perceived personal relevance.

Teachers also identified several potential benefits of integrating mindfulness into educational contexts, particularly for students’ emotional wellbeing. Some emphasized its role in reducing language anxiety, noting that “*students may hesitate or feel embarrassed when they are inadequate in speaking a foreign language… mindfulness practices can be used to alleviate these feeling*s *(Interviewee 5)*.” Others highlighted its usefulness in addressing exam stress: “*If we were to introduce these techniques to eighth-grade students, I would use them to relieve stress… especially to help them cope with exam anxiety*. *(Interviewee 7)*” These excerpts suggest that teachers perceive mindfulness as a practical tool for addressing common emotional challenges in the classroom.

However, these perceived benefits were accompanied by concerns about feasibility. Teachers questioned how mindfulness practices could be incorporated into already structured lessons without disrupting instructional flow. One participant explained, “*It’s hard to come back… it might be difficult for students to refocus on the lesson*. *(Interviewee 5)*” Additionally, concerns about institutional perceptions were prominent. As one teacher noted, “*The administration might think the teacher is acting like a guidance counselor… Parents might say: ‘Are they teaching or meditating?*’ *(Interviewee 6)*” These comments highlight a perceived lack of legitimacy for mindfulness within the current educational culture, which may discourage its implementation. Participants also identified several barriers, including time constraints, heavy workloads, lack of training, and uncertainty about the application. Time limitations were frequently mentioned, with one teacher stating, “*Considering the limited amount of time we have, it becomes an obstacle to frequently using mindfulness practices*. *(Interviewee 3)*” Similarly, lack of training contributed to hesitation: “*I don’t know how to implement it, so I would like to learn*. *(Interviewee 4)*” Some participants also expressed concerns about overuse, with one cautioning, “*If I use it too much, it might affect the children… repetition could lead to negative outcomes*. *(Interviewee 2)*” These reflections demonstrate that teachers critically evaluate new practices rather than passively accepting them. Importantly, these barriers were often accompanied by a willingness to engage under appropriate conditions. As one teacher noted, “*If a seminar were provided on how to effectively implement mindfulness practices, it would influence my use*. *(Interviewee 8)*” This suggests that reluctance is not rooted in rejection but in a lack of confidence and structural support.

## 4 Discussion

This pilot study aimed to provide a context-sensitive foundation for the design of a mindfulness-informed professional development (PD) intervention for EFL teachers. While the findings partially align with existing literature, a closer examination reveals important contextual differences that can be attributed to structural, cultural, and educational characteristics of the Turkish EFL context. More importantly, this study extends prior work by explicitly linking empirical findings to concrete intervention design decisions.

The quantitative findings indicated that teachers demonstrated moderate overall mindfulness, with stronger skills in observing and describing and weaker performance in acting with awareness and accepting without judgment. This pattern is consistent with previous studies (Baer et al., 2004; Dekeyser et al., 2008) suggesting that attentional noticing and verbal labeling are more accessible than sustained attention and non-judgmental awareness. One explanation is that observing and describing are already embedded in teaching practices, such as monitoring classroom interactions and reflecting on student behavior. In contrast, acting with awareness and accepting without judgment require more advanced regulatory capacities that are not typically developed through conventional teacher education. However, compared to intervention-based studies (e.g., Flook et al., 2013; Jennings, 2015), participants in this study demonstrated lower competence in higher-order mindfulness skills, particularly acceptance. This difference can be explained by the absence of structured mindfulness training in the current context. In educational systems where mindfulness is integrated into teacher education or school-based programs (e.g., Harris et al., 2016; Hatton-Bowers et al., 2020), teachers are more likely to develop these competencies. In contrast, participants in this study operate within a system where emotional demands are high, but systematic support for emotional regulation is limited. This gap likely contributes to the imbalance observed between foundational and more complex mindfulness skills.

The qualitative findings revealed limited conceptual understanding of mindfulness among teachers, which contrasts with studies conducted in contexts where mindfulness is already part of professional discourse. This uncertainty appears to stem from a lack of exposure. Teachers often constructed their understanding during the interviews, suggesting that mindfulness is experienced as an externally introduced concept rather than an established professional competency. This finding has direct implications for intervention design. The forthcoming program will include a structured psychoeducational component aimed at establishing a clear and contextually relevant understanding of mindfulness. This component will explicitly position mindfulness as a professional resource for managing classroom challenges, rather than as a purely therapeutic or abstract construct. By grounding mindfulness in everyday teaching practices, the intervention seeks to enhance its legitimacy and accessibility.

Teachers’ perceptions of the potential benefits of mindfulness—particularly in relation to emotional regulation, stress reduction, and student anxiety—are consistent with prior research (Albrecht et al., 2012; Gouda et al., 2016; Huang, 2022). Participants recognized its potential to support both teacher wellbeing and classroom climate. However, these positive perceptions coexisted with concerns about feasibility. Teachers expressed uncertainty about how mindfulness practices could be integrated into tightly structured lessons without disrupting instructional flow or attracting negative attention from administrators and parents. These concerns can be understood in relation to the broader institutional context, which is characterized by time constraints, exam-oriented curricula, and performance-based expectations. In such environments, practices perceived as non-instructional may be viewed as inappropriate or inefficient. This contrasts with findings from contexts where mindfulness is already normalized within school systems (Kenwright et al., 2023). Therefore, feasibility concerns in this study are not merely individual but reflect systemic constraints. In response, the intervention will prioritize the integration of brief, classroom-compatible mindfulness practices. Rather than introducing separate sessions, the program will embed short (3–5 min) activities into existing instructional routines. These practices will be explicitly linked to pedagogical goals, such as reducing speaking anxiety or enhancing student focus. This approach aims to address teachers’ concerns about time, legitimacy, and instructional continuity.

The finding that observing was the strongest predictor of overall mindfulness provides an important basis for intervention design. Previous research (Shapiro et al., 2006) suggests that awareness-based practices can facilitate the development of more complex mindfulness skills. In this study, teachers’ relatively strong observational abilities likely reflect their professional experience in attending to classroom dynamics. However, without structured support, these skills do not automatically translate into emotional regulation or non-judgment. Accordingly, the intervention will adopt a scaffolded, phase-based structure. The initial phase will focus on strengthening awareness through observation-based practices, such as breath awareness and sensory noticing. The second phase will extend this awareness into teaching practice, supporting teachers in maintaining attention during instructional tasks. The final phase will target emotional regulation and acceptance, introducing strategies for responding to challenging classroom situations with reduced reactivity. This progression reflects both empirical findings and theoretical models of mindfulness development, while remaining sensitive to participants’ starting points.

Teachers expressed a preference for practical, flexible, and contextually relevant professional development. While previous studies often emphasize the importance of consistent daily practice (Gold et al., 2010; Harris et al., 2016), participants in this study raised concerns about time constraints and potential practice fatigue. These concerns highlight the need to adapt intervention intensity to the realities of teachers’ working conditions. Therefore, the forthcoming intervention will adopt a low-burden engagement model. Practices will be brief, adaptable, and optional, allowing teachers to integrate them into their routines without excessive demand. This approach prioritizes sustainability and teacher autonomy, which are critical for long-term engagement in resource-constrained contexts.

While the findings broadly align with existing research on teacher mindfulness, the differences observed in this study can be explained by contextual factors including limited exposure, institutional expectations, and cultural perceptions of mindfulness. By explicitly examining these factors, this study moves beyond surface-level comparison and provides a context-sensitive framework for intervention design. The forthcoming mindfulness-informed PD intervention will (1) provide foundational psychoeducation to address conceptual gaps, (2) scaffold mindfulness skills from observation to acceptance, (3) integrate brief, curriculum-aligned practices to enhance feasibility, and (4) address institutional and cultural barriers to support implementation. In doing so, the study contributes to the development of context-sensitive mindfulness interventions. Besides, this study extends the literature by highlighting the cognitive and positive psychological factors that shape language teachers’ professional experiences, including mindfulness, emotional regulation, attentional awareness, and wellbeing. By contextualizing these factors within Turkish EFL classrooms, the study contributes to a deeper understanding of how psychological resources influence teachers’ resilience, instructional responsiveness, and professional development. It adds to the recent research on the psychological aspects of language teaching (Bardach et al., 2024; de Vries et al., 2013; Mohammadzadeh Mohammadabadi, 2025) provides practical insights for designing context-sensitive mindfulness-informed professional development programs for EFL teachers.

## Data Availability

The original contributions presented in this study are included in this article/Supplementary material, further inquiries can be directed to the corresponding author.

## References

[B1] Albrecht N. J. Albrecht P. Cohen M. (2012). Mindfully teaching in the classroom: A literature review. *Aust. J. Teach. Educ.* 37 1–14. 10.14221/ajte.2012v37n12.2

[B2] Baer R. A. Smith G. T. Allen K. B. (2004). Assessment of mindfulness by self-report: The Kentucky inventory of mindfulness skills. *Assessment* 11 191–206. 10.1177/1073191104268029 15358875

[B3] Bardach L. Bostwick K. Fütterer T. Kopatz M. Daniel Memarpour Hobbi, Klassen R. M. et al. (2024). A meta-analysis on teachers’ growth mindset. *Educ. Psychol. Rev.* 36:84. 10.1007/s10648-024-09925-7

[B4] Birchinall L. Spendlove D. Buck R. (2019). In the moment: Does mindfulness hold the key to improving the resilience and well-being of pre-service teachers? *Teach. Teach. Educ.* 86:102919. 10.1016/j.tate.2019.102919

[B5] Buchanan T. K. (2017). Mindfulness and meditation in education. *Young Child.* 72 69–74.

[B6] Cicek M. Gurbuz N. (2023). Exploring the impacts of mindfulness training for an EFL teacher: Insights from a narrative inquiry study. *Issues Educ. Res.* 33 471–487.

[B7] Clarke J. Draper S. (2020). Intermittent mindfulness practice can be beneficial, and daily practice can be harmful: An in-depth, mixed methods study of the “Calm” app’s (mostly positive) effects. *Internet Interv.* 19 1–11. 10.1016/j.invent.2019.100293 31890639 PMC6928287

[B8] Corthorn C. Pedrero V. Torres N. Reynaldos-Grandón K. Paredes P. (2024). Mindfulness, teacher mental health, and well-being in early education: A correlational study. *BMC Psychol.* 12:428. 10.1186/s40359-024-01930-3 39107861 PMC11304776

[B9] Creswell J. W. Creswell J. D. (2017). *Research Design: Qualitative, Quantitative, and Mixed Methods Approaches*, 5th Edn. London: Sage Publications.

[B10] Dave D. J. McClure L. A. Rojas S. R. De Lavalette O. Lee D. J. (2020). Impact of mindfulness training on the well-being of educators. *J. Altern. Complement. Med.* 26 645–651. 10.1089/acm.2019.0451 32453627

[B11] de Vries S. Jansen E. P. van de Grift W. J. (2013). Profiling teachers’ continuing professional development and the relation with their beliefs about learning and teaching. *Teach. Teach. Educ.* 33 78–89. 10.1016/j.tate.2013.02.006

[B12] Dekeyser M. Raes F. Leijssen M. Leysen S. Dewulf D. (2008). Mindfulness skills and interpersonal behaviour. *Pers. Individ. Dif.* 44 1235–1245. 10.1016/j.paid.2007.11.018

[B13] Demir B. Hamarat B. Sönmez G. (2025). Exploring the role of well-being, mindfulness, life satisfaction, and subjective happiness in the professional development of language teachers. *Psychol. Sch.* 62 1641–1658. 10.1002/pits.23420

[B14] Derakhshan A. (2022). Revisiting research on positive psychology in second and foreign language education: Trends and directions. *Lang. Relat. Res.* 13 1–43. 10.52547/LRR.13.5.2

[B15] DeVellis R. F. (2017). *Scale Development: Theory and Applications*, 4th Edn. London: Sage Publications.

[B16] DiCarlo C. F. Meaux A. B. LaBiche E. H. (2020). Exploring mindfulness for perceived teacher stress and classroom climate. *Early Child. Educ. J.* 48 485–496. 10.1007/s10643-019-01015-6

[B17] Field A. (2018). *Discovering Statistics Using IBM SPSS Statistics*, 5th Edn. London: Sage.

[B18] Flook L. Goldberg S. B. Pinger L. Bonus K. Davidson R. J. (2013). Mindfulness for teachers: A pilot study to assess effects on stress, burnout, and teaching efficacy. *Mind Brain Educ.* 7 182–195. 10.1111/mbe.12026 24324528 PMC3855679

[B19] Frank J. L. Jennings P. A. Greenberg M. T. (2016). Validation of the mindfulness in teaching scale. *Mindfulness* 7, 155–163. 10.1007/s12671-015-0461-0

[B20] Gold E. Smith A. Hopper I. Herne D. Tansey G. Hulland C. (2010). Mindfulness-based stress reduction (MBSR) for primary school teachers. *J. Child Fam. Stud.* 19 184–189. 10.1007/s10826-009-9344-0

[B21] Gouda S. Luong M. T. Schmidt S. Bauer J. (2016). Students and teachers benefit from mindfulness-based stress reduction in a school-embedded pilot study. *Front. Psychol.* 7:590. 10.3389/fpsyg.2016.00590 27199825 PMC4845593

[B22] Hair J. F. Anderson R. E. Tatham R. L. Black W. C. (2013). *Multivariate Data Analysis*, 7th Edn. Pearson.

[B23] Harris A. R. Jennings P. A. Katz D. A. Abenavoli R. M. Greenberg M. T. (2016). Promoting stress management and wellbeing in educators: Feasibility and efficacy of a school-based yoga and mindfulness intervention. *Mindfulness* 7 143–154.

[B24] Hatton-Bowers H. Howell Smith M. Huynh T. Bash K. Durden T. Anthony C. et al. (2020). “I will be less judgmental, more kind, more aware, and resilient!”: Early childhood professionals’ learnings from an online mindfulness module. *Early Child. Educ. J.* 48 379–391. 10.1007/s10643-019-01007-6

[B25] He J. Iskhar S. Yang Y. Aisuluu M. (2023). Exploring the relationship between teacher growth mindset, grit, mindfulness, and EFL teachers’ well-being. *Front Psychol.* 14:1241335. 10.3389/fpsyg.2023.1241335 37818422 PMC10561394

[B26] Henriksen D. Gruber N. Woo L. (2022). Looking to the future from the present: A call for well-being and mindfulness in education. *J. Technol. Teach. Educ.* 30 145–154. 10.70725/291616uhregq

[B27] Hidajat T. J. Edwards E. J. Wood R. Campbell M. (2023). Mindfulness-based interventions for stress and burnout in teachers: A systematic review. *Teach. Teach. Educ.* 134:104303. 10.1016/j.tate.2023.104303

[B28] Hoskins C. J. (2020). *The Relationship Between Mindfulness and Feelings of Perceived Job Intensification in Secondary Teachers (Publication No. 28088721).* Doctoral dissertation, ProQuest Dissertations & Theses Global: Ann Arbor, MI.

[B29] Huang J. (2022). The Role of English as a Foreign Language teachers’ mindfulness and compassion in fostering students’ foreign language enjoyment. *Front. Psychol.* 13:899298. 10.3389/fpsyg.2022.899298 35592155 PMC9110884

[B30] Hue M. Lau N. (2015). Promoting well-being and preventing burnout in teacher education: A pilot study of a mindfulness-based programme for pre-service teachers in Hong Kong. *Teach. Dev.* 19 381–401. 10.1080/13664530.2015.1049748

[B31] Jennings P. A. (2015). Early childhood teachers’ well-being, mindfulness, and self-compassion in relation to classroom quality and attitudes towards challenging students. *Mindfulness* 6 732–743. 10.1007/s12671-014-0312-4

[B32] Kabat-Zinn J. (2005). *Coming to Our Senses: Healing Ourselves and the World Through Mindfulness.* London: Hachette UK.

[B33] Kabat-Zinn J. (2015). Mindfulness. *Mindfulness* 6 1481–1483. 10.1007/s12671-015-0456-x

[B34] Kenwright D. McLaughlin T. Hansen S. (2023). Teachers ’perspectives about mindfulness programmes in primary schools to support wellbeing and positive behaviour. *Int. J. Incl. Educ.* 27 739–754. 10.1080/13603116.2020.1867382

[B35] Khodabakhshzadeh H. Esmaeilnia M. Sarani L. Gholami F. (2020). Examining the relationship between the level of mindfulness and job anxiety among EFL teachers in Iran. *J. Appl. Linguist. Lang. Res.* 7 79–86.

[B36] Koçali Z. Asik A. (2022). A systematic review of mindfulness studies in ESL and EFL contexts. *Imanagers J. Educ. Psychol.* 15 47–61. 10.26634/jpsy.15.3.18588

[B37] Kunselman A. R. (2024). A brief overview of pilot studies and their sample size justification. *Fertil. Steril.* 121, 899–901. 10.1016/j.fertnstert.2024.01.040 38331310 PMC11128343

[B38] Kuru Gönen S. İ. (2022). Mindfulness-based practices for EFL teachers: Sample tasks and insights to cultivate mindfulness. *Focus ELT J.* 4. 10.14744/felt.2022.4.3.6

[B39] Kyte D. (2016). Toward a sustainable sense of self in teaching and teacher education: Sustainable happiness and well-being through mindfulness. *Mcgill J. Educ.* 51 1143–1162. 10.7202/1039632ar

[B40] Lomas T. Medina J. C. Ivtzan I. Rupprecht S. Eiroa-Orosa F. J. (2017). The impact of mindfulness on the well-being and performance of educators: A systematic review of the empirical literature. *Teach. Teach. Educ.* 61 132–141. 10.1016/j.tate.2016.10.008

[B41] Mohammadzadeh Mohammadabadi A. (2025). Profiling language teachers’ implicit theories of the future: Future mindsets as predictors of language teachers’ emotions. *Lang. Teach. Futur.* 1 6–22.

[B42] Molloy Elreda L. Jennings P. A. DeMauro A. A. Mischenko P. P. Brown J. L. (2019). Protective effects of interpersonal mindfulness for teachers’ emotional supportiveness in the classroom. *Mindfulness* 10 537–546. 10.1007/s12671-018-0996-y

[B43] Nadlifah K. Setiawan S. Munir A. (2023). Positive psychology to flourish professional well-being: A qualitative study of Indonesian English teachers’ perspective. *JEES* 8 85–94. 10.21070/jees.v8i1.1693

[B44] Pan M. Liu J. (2022). Chinese English as a Foreign Language Teachers’ wellbeing and motivation: The role of mindfulness. *Front. Psychol.* 13:906779. 10.3389/fpsyg.2022.906779 35664142 PMC9161141

[B45] Roeser R. W. Skinner E. Beers J. Jennings P. A. (2012). Mindfulness training and teachers’ professional development: An emerging area of research and practice. *Child Dev. Perspect.* 6 167–173. 10.1111/j.1750-8606.2012.00238.x

[B46] Shapiro S. L. Carlson L. E. Astin J. A. Freedman B. (2006). Mechanisms of mindfulness. *J. Clin. Psychol.* 62 373–386. 10.1002/jclp.20237 16385481

[B47] Skelly K. J. Estrada-Chichón J. L. (2021). Mindfulness as a coping strategy for EFL learning in education. *Int. J. Instruct.* 14 965–980. 10.29333/iji.2021.14356a

[B48] Song X. He X. (2021). Teachers’ dispositions toward mindfulness in EFL/ESL classrooms in teacher-student interpersonal relationships. *Front. Psychol.* 12:754998. 10.3389/fpsyg.2021.754998 34603174 PMC8481581

[B49] Takiguchi H. (2015). Implementing mindfulness into teaching English as a second language at the college level: An exploratory study. *Departmental Bull. Pap.* 50, 127–142. 10.15004/00001395

[B50] Teddlie C. Tashakkori A. (2009). *Foundations of Mixed Methods Research: Integrating Quantitative and Qualitative Approaches in the Social and Behavioral Sciences.* London: SAGE Publications.

[B51] Tuyan S. Kabadayi B. (2018). “Cultivating mindfulness in the EFL classroom: An exploratory study,” in *Empowering Teacher Researchers, Empowering Learners*, eds Barkhuizen G. Burns A. Kenan D. Wyatt M. (Harrogate: IATEFL), ∙ 67–73.

[B52] Wanders F. H. Dijkstra A. B. Maslowski R. Van der Veen I. (2020). The effect of teacher-student and student-student relationships on the societal involvement of students. *Res. Pap. Educ.* 35 266–286. 10.1080/02671522.2019.1568529

[B53] Wang N. (2022). EFL teachers’ mindfulness and emotion regulation in language context. *Front. Psychol.* 13:877108. 10.3389/fpsyg.2022.877108 35756308 PMC9221676

[B54] Wang Y. Liu C. (2016). Cultivate mindfulness: A case study of mindful learning in an English as a foreign language classroom. *IAFOR J. Educ.* 4 141–155. 10.22492/ije.4.2.08

[B55] Weisi H. Salari M. (2024). On the journey from cognizance toward thriving: Iranian EFL teachers’ engagement in reflective practice and professional development: The mediating effect of teacher mindfulness. *Reflect. Pract.* 25 550–564. 10.1080/14623943.2024.2370596

[B56] Xu Y. Wang J. (2024). The mediating role of teaching enthusiasm in the relationship between mindfulness, growth mindset, and psychological well-being of Chinese EFL teachers. *Humanit. Soc. Sci. Commun.* 11:1176. 10.1057/s41599-024-03694-y

[B57] Zhi R. Wang Y. (2024). Exploring the interplay between well-being, mindfulness, creativity, and work engagement among EFL teachers. *Think. Skills Creat.* 56:101697. 10.1016/j.tsc.2024.101697

[B58] Zhu D. (2022). English as a Foreign Language teachers’ identity and motivation: The role of mindfulness. *Front. Psychol.* 13:940372. 10.3389/fpsyg.2022.940372 35837643 PMC9274092

