# Supplementary material for: A pilot study on integrating mindfulness-informed professional development for EFL teachers
Source: Front Psychol. 2026 Jun 11;17:1771786. doi: 10.3389/fpsyg.2026.1771786 (PMC13293943; doi:10.3389/fpsyg.2026.1771786)
Supplement: Supplementary file 1 [file Table_1.DOCX]

Supplementary Material

**
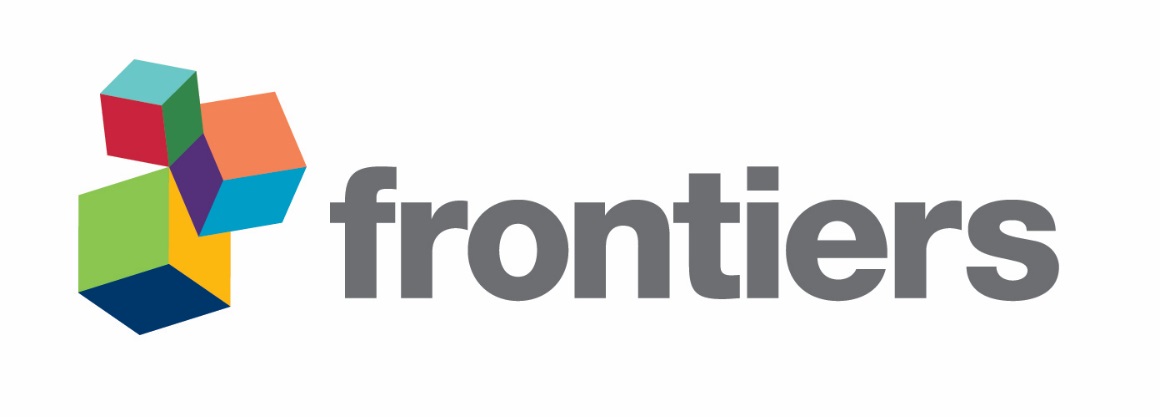
**

**Supplementary Table 1.** Descriptive Statistics for the Mindfulness in Teaching Scale (MTS)

| *Items in the Scale* | M | SD |
| --- | --- | --- |
| 1. I notice changes in my body, such as whether my breathing slows down or speeds up | 4.00 | .894 |
| 2. I’m good at finding the words to describe my feelings | 4.35 | .877 |
| 3. When I do things, my mind wanders off and I’m easily distracted | 4.35 | .755 |
| 4. When I am teaching I get so focused on the goal I want to achieve that I lose touch with what I’m doing right now to get there | 4.06 | 1.093 |
| 5. At school I tend to walk quickly to get where I’m going without paying attention to what I experience along the way | 3.97 | 1.140 |
| 6. I rush through activities with my class without being really attentive to them | 4.10 | 1.012 |
| 7. When something painful happens at school I tend to blow the incident out of proportion | 4.13 | 1.024 |
| 8. I am often so busy thinking about other things that I am not really listening to my students | 4.29 | .864 |
| 9. When I’m really struggling with teaching, I tend to feel like other teachers must be having an easier time of it | 3.48 | 1.029 |
| 10. Even when it makes me uncomfortable, I allow my students to express their feelings | 3.42 | 1.232 |
| 11. I listen carefully to my student’s ideas, even when I disagree with them | 4.26 | .729 |
| 12. I am aware of how my moods affect the way I treat my students | 3.97 | 1.016 |
| 13. When I’m upset with my students, I notice how I am feeling before I take action | 3.77 | .805 |
| 14. When I am upset with my class, I calmly tell them how I am feeling | 3.52 | .926 |
