# Supplementary material for: A pilot study on integrating mindfulness-informed professional development for EFL teachers
Source: Front Psychol. 2026 Jun 11;17:1771786. doi: 10.3389/fpsyg.2026.1771786 (PMC13293943; doi:10.3389/fpsyg.2026.1771786)
Supplement: Supplementary file 2 [file Table_2.DOCX]

Supplementary Material

**
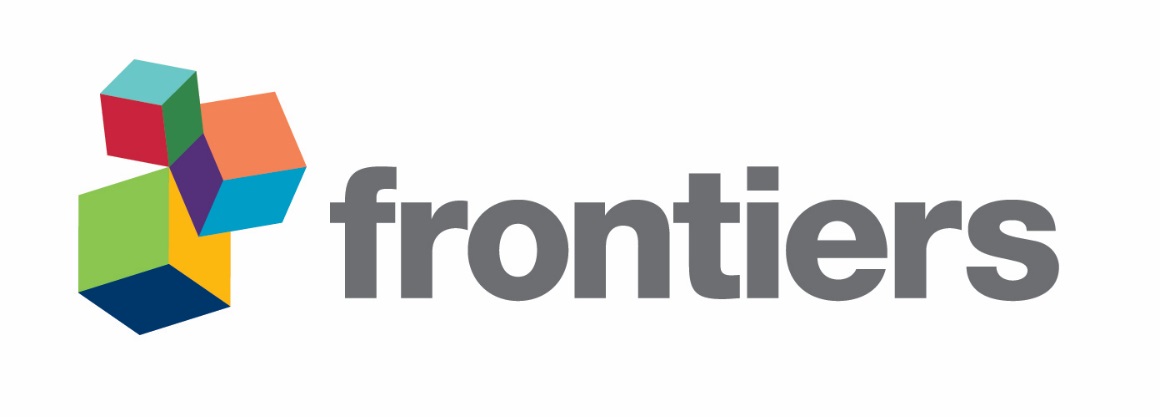
**

**Supplementary Table 2.** Descriptive Statistics for English Teachers' Observation Dimension

| *Items in the Scale* | *M* | *SD* |
| --- | --- | --- |
| 1. I notice changes in my body, such as whether my breathing slows down or speeds up | 3.48 | 1.029 |
| 5. I pay attention to whether my muscles are tense or relaxed | 2.90 | 1.106 |
| 9. When I’m walking, I deliberately notice the sensations of my body moving | 3.29 | .973 |
| 13. When I take a shower or bath, I stay alert to the sensations of water on my body | 3.00 | 1.155 |
| 17. I notice how foods and drinks affect my thoughts, bodily sensations, and emotions | 3.00 | 1.342 |
| 21. I pay attention to sensations, such as the wind in my hair or sun on my face | 3.13 | 1.118 |
| 25. I pay attention to sounds, such as clocks ticking, birds chirping, or cars passing | 3.48 | 1.180 |
| 29. I notice the smells and aromas of things | 3.61 | .803 |
| 30. I intentionally stay aware of my feelings | 2.81 | 1.223 |
| 33. I notice visual elements in art or nature, such as colors, shapes, textures, or patterns of light and shadow | 3.84 | .860 |
| 37. I pay attention to how my emotions affect my thoughts and behavior | 3.94 | .854 |
| 39. I notice when my moods begin to change | 3.84 | .969 |
