# Supplementary material for: A pilot study on integrating mindfulness-informed professional development for EFL teachers
Source: Front Psychol. 2026 Jun 11;17:1771786. doi: 10.3389/fpsyg.2026.1771786 (PMC13293943; doi:10.3389/fpsyg.2026.1771786)
Supplement: Supplementary file 3 [file Table_3.DOCX]

Supplementary Material

**
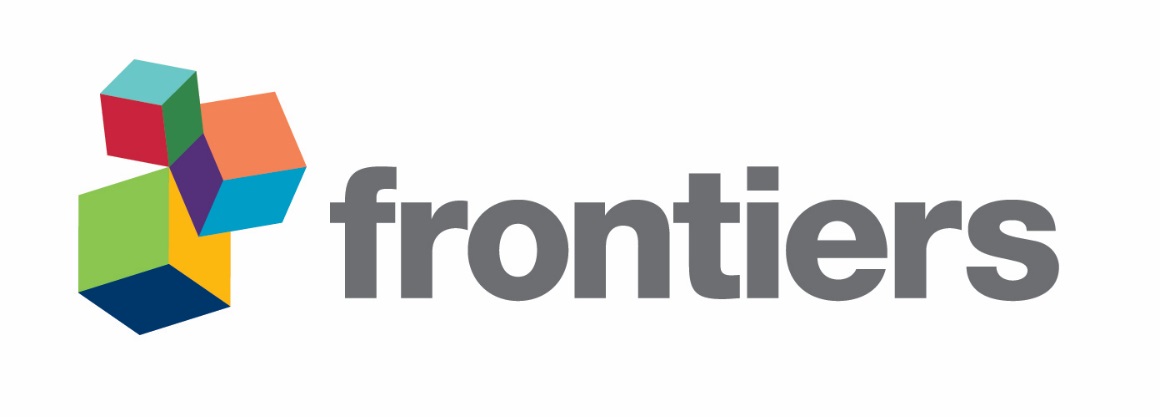
**

**Supplementary Table 3.** Descriptive Statistics for English Teachers' Description Dimension

| *Items in the Scale* | *M* | *SD* |
| --- | --- | --- |
| 2. I’m good at finding the words to describe my feelings | 3.81 | ,792 |
| 6. I can easily put my beliefs, opinions, and expectations into words | 3.74 | .999 |
| 10. I’m good at thinking of words to express my perceptions, such as how things taste, smell, or sound | 3.97 | .752 |
| 14. It’s hard for me to find the words to describe what I’m thinking | 2.16 | .934 |
| 18. I have trouble thinking of the right words to express how I feel about things | 2.10 | .978 |
| 22. When I have a sensation in my body, it’s difficult for me to describe it because I can’t find the right words | 2.00 | .966 |
| 26. Even when I’m feeling terribly upset, I can find a way to put it into words | 3.71 | .693 |
| 34. My natural tendency is to put my experiences into words | 3.58 | .923 |
