# Supplementary material for: A pilot study on integrating mindfulness-informed professional development for EFL teachers
Source: Front Psychol. 2026 Jun 11;17:1771786. doi: 10.3389/fpsyg.2026.1771786 (PMC13293943; doi:10.3389/fpsyg.2026.1771786)
Supplement: Supplementary file 4 [file Table_4.DOCX]

Supplementary Material

**
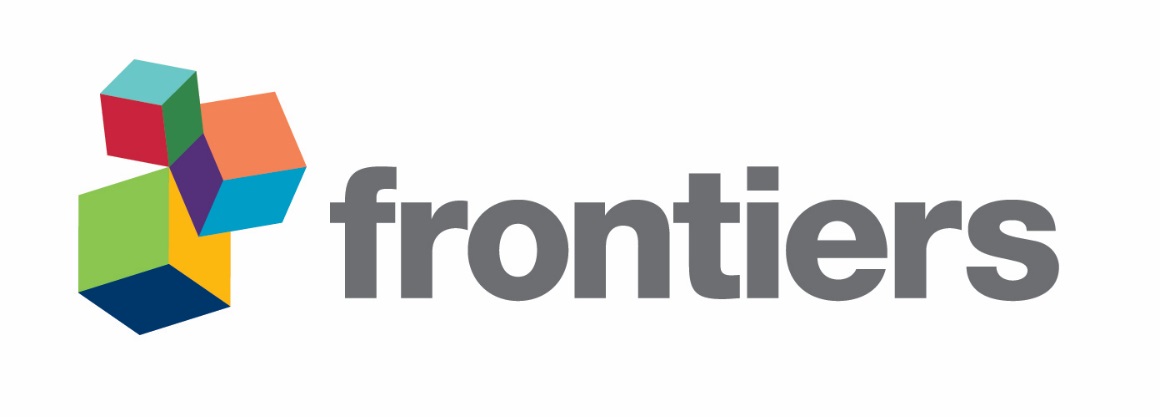
**

**Supplementary Table 4.** Descriptive Statistics for English Teachers' Acting with Awareness Dimension

| *Items in the Scale* | *M* | *SD* |
| --- | --- | --- |
| 3. When I do things, my mind wanders off and I’m easily distracted | 3.32 | 1.045 |
| 7. When I’m doing something, I’m only focused on what I’m doing, nothing else | 3.06 | 1.237 |
| 11. I drive on “automatic pilot” without paying attention to what I’m doing | 4.03 | .983 |
| 15. When I’m reading, I focus all my attention on what I’m reading | 3.10 | 1.136 |
| 19. When I do things, I get totally wrapped up in them and don’t think about anything else | 2.55 | .995 |
| 23. I don’t pay attention to what I’m doing because I’m daydreaming, worrying, or otherwise distracted | 4.10 | .944 |
| 27. When I’m doing chores, such as cleaning or laundry, I tend to daydream or think of other things | 2.65 | 1.170 |
| 31. I tend to do several things at once rather than focusing on one thing at a time | 2.81 | 1.223 |
| 35. When I’m working on something, part of my mind is occupied with other topics, such as what I’ll be doing later, or things I’d rather be doing | 2.71 | 1.006 |
| 38. I get completely absorbed in what I’m doing, so that all my attention is focused on it | 3.06 | .929 |
