# Supplementary material for: A pilot study on integrating mindfulness-informed professional development for EFL teachers
Source: Front Psychol. 2026 Jun 11;17:1771786. doi: 10.3389/fpsyg.2026.1771786 (PMC13293943; doi:10.3389/fpsyg.2026.1771786)
Supplement: Supplementary file 5 [file Table_5.DOCX]

Supplementary Material

**
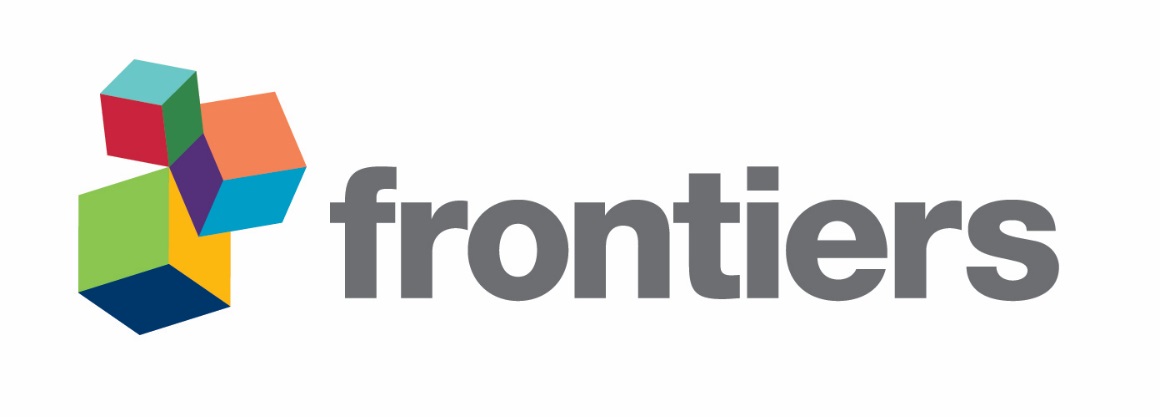
**

**Supplementary Table 5.** Descriptive Statistics for English Teachers' Acting without Judgment Dimension

| *Items in the Scale* | *M* | *SD* |
| --- | --- | --- |
| 4. I criticize myself for having irrational or inappropriate emotions | 2.58 | 1.025 |
| 8. I tend to evaluate whether my perceptions are right or wrong | 2.32 | .702 |
| 12. I tell myself that I shouldn’t be feeling the way I’m feeling | 2.90 | 1.193 |
| 16. I believe some of my thoughts are abnormal or bad and I shouldn’t think that way | 3.45 | 1.121 |
| 20. I make judgments about whether my thoughts are good or bad | 2.68 | 1.013 |
| 24. I tend to make judgments about how worthwhile or worthless my experiences are | 2.81 | 1.167 |
| 28. I tell myself that I shouldn’t be thinking the way I’m thinking | 3.19 | 1.078 |
| 32. I think some of my emotions are bad or inappropriate and I shouldn’t feel them | 3.23 | 1.023 |
| 36. I disapprove of myself when I have irrational ideas | 2.90 | .978 |
